# Study on the Antioxidant Activity of Fermented Broad Bean–Mulberry Composite Juice Based on In Vitro Digestion and Non-Targeted Metabolomic Analysis

**DOI:** 10.3390/foods15060991

**Published:** 2026-03-11

**Authors:** Yue Zhao, Weiqiao Pang, Ying Wang, Wei Sun, Ruinan Gao, Zili Zhao, Bing Li

**Affiliations:** 1 College of Food Science, Heilongjiang Bayi Agricultural University, Daqing 163319, China; zhaoyue03232024@163.com (Y.Z.); swsw0102@163.com (W.S.); gaoruinan0459@163.com (R.G.); 2 College of Biological Engineering, Daqing Normal University, Daqing 163319, China; hljbypwq@163.com; 3 National Coarse Cereals Engineering Research Center, Daqing 163319, China; 4 College of Food Science, Xiamen Ocean Vocational College, Xiamen 361100, China; 5 Anhui Academy of Agricultural Sciences Northern Anhui Research Institute, Hefei 236658, China; zhaozili1965@163.com (Z.Z.); libing2504@sina.com (B.L.)

**Keywords:** fermented composite juice, in vitro simulated digestion, antioxidant activity, non-targeted metabolomics

## Abstract

Fermentation is a widely utilized technology that efficiently enriches bioactive compounds, thereby enhancing the bioactivity of food. This study aimed to investigate the release of the total polyphenol content (TPC) and flavonoid content (TFC), changes in antioxidant activity, and in vitro relative abundance trends of phenolic metabolites in the fermented composite juice of kidney beans and mulberries. An in vitro simulated gastric and intestinal digestion method was employed to examine the release patterns of polyphenols and flavonoids, along with alterations in antioxidant activity during the gastrointestinal digestion of the fermented composite juice. Non-targeted metabolomics LC-MS technology was employed to detect changes in the relative abundance and enrichment of phenolic metabolites during fermentation and digestion stages. The results indicated that after simulated digestion, the polyphenol content increased by 1.42-fold and the flavonoid content by 2.53-fold compared to pre-digestion. The scavenging rates for DPPH radicals, hydroxyl radicals, and ABTS radicals reached 85.44%, 94.77%, and 76.12%, respectively. Non-targeted metabolomic investigation through KEGG pathway enrichment showed associations between phenolic metabolites and antioxidant activity. During fermentation and digestion, daidzein, genistein, quercetin, and catechin may be the potential compounds contributing to the enhanced antioxidant activity of FBMJ. Among these, phenolic metabolites are significantly enriched in the biosynthetic pathways of flavonoids and flavanols. This study has elucidated the metabolic variations between the fermentation and digestion stages of fermented composite juice from a metabolomics perspective, providing preliminary in vitro research evidence and theoretical clues for developing it as a functional food with antioxidant potential.

## 1. Introduction

Kidney beans (*Phaseolus vulgaris* L.), commonly referred to as common beans, are a significant crop within the Phaseolus genus of the Fabaceae family. Their cultivation is widespread in China, making them the second most cultivated edible legume crop after soybeans. China is among the world’s leading producers and exporters of kidney beans, with Heilongjiang Province accounting for approximately half of the nation’s annual exports, thereby demonstrating a notable regional advantage [1]. Kidney beans are abundant in bioactive components, including polyphenols and flavonoids, which effectively scavenge free radicals and enhance the body’s antioxidant capacity [2]. Furthermore, their high biological activity promotes microbial growth, making fermentation an effective method to enhance the nutritional value of the final product. On the other hand, mulberries (Fructus mori), also known as mulberry fruit, are a highly nutrient-dense plant with applications in both food and medicine [3]. They are rich in phenolic compounds, including flavonoids, phenolic acids, and anthocyanins, which exhibit significant antioxidant properties [4]. Additionally, their high sugar content makes fermented mulberries an excellent carbon source for lactic acid bacteria, facilitating more thorough fermentation. Wang et al. [5] utilized lactic acid fermentation to enhance the antioxidant potential, as well as the polyphenol and flavonoid contents, of kidney beans and blueberries. Consequently, combining kidney beans with mulberries to create a composite fermented juice could enhance its functional activity. However, the activity and transformation patterns of bioactive components, such as polyphenols, during fermentation and subsequent to ingestion remain poorly understood, significantly limiting their practical applications.

An essential technique for examining the absorption and digestion of active ingredients is in vitro simulated digestion [6]. In this study, probiotic fermentation was employed to enhance bacterial survival rates under acidic conditions, while simultaneously ensuring better retention of active components in the raw materials during simulated digestion. Julianna et al. [7] analyzed the aqueous and ethanolic extracts of fruit residues regarding assessment of the α-amylase inhibition, cytotoxicity, and bioaccessibility of phenolic compounds, flavonoids and antioxidants after in vitro gastrointestinal digestion and fermentation with probiotic compounds by UFLC-DAD. Furthermore, metabolomics has been applied to examine the alterations of phenolic compounds during digestion, facilitating the precise identification of their evolution in food throughout specific stages of fermentation or processing, as well as the investigation of potential metabolic pathways involved [8]. Specifically, non-targeted metabolomics is a more comprehensive approach for detecting and identifying all small-molecule metabolites in biological samples, thereby enhancing the study of metabolic processes by comparing metabolic variations across different groups [9]. Chen et al. [10] investigated the hypoglycemic activity of polyphenols derived from purple laver at various harvest times using in vitro simulated digestion and non-targeted metabolomics. Additionally, Serena et al. [11] assessed the intestinal bioavailability of oat beverages through non-targeted metabolomics following lactobacillus fermentation and in vitro digestion simulation. In summary, this study utilizes non-targeted metabolomics to comprehensively screen significant substances involved in sample metabolism, shedding light on their metabolic differences and pathways. Currently, few studies employ non-targeted metabolomics to analyze the fermentation and digestion processes of fermented composite juices, with most research focusing solely on metabolic variations during fermentation.

In this study, we investigated the changes in the polyphenol content (TPC) and flavonoid content (TFC) and antioxidant activity of fermented composite juice. Utilizing an in vitro simulated digestion model, we analyzed the variations in the TPC and TFC, as well as antioxidant activity, during the digestion process of fermented composite juice prepared from kidney beans and mulberries. To give a thorough grasp of the relevant metabolic processes, we utilized liquid chromatography–mass spectrometry (LC-MS) non-targeted metabolomics to compare phenolic compound abundance at various stages: pre- and post-fermentation, gastric digestion, and intestinal digestion. This research aims to explore the antioxidant mechanisms in fermented fruit juices and establish a theoretical foundation for future functional studies, thereby establishing the theoretical groundwork for the industrial development and use of fermented kidney bean–mulberry composite juices in the future.

## 2. Materials and Methods

### 2.1. Materials and Reagents

Black kidney beans and mulberry puree were obtained from the Heilongjiang reclamation area (Heilongjiang, China). High-temperature-resistant α-amylase (20,000 U/mL) was bought from Maclean Biochemical Technology Co., Ltd. (Shanghai, China). Amylase (10,000 U/mL) and pectinase (500 U/mg) were purchased from Sigma-Aldrich Co. Ltd. (St. Louis, MO, USA). LP (*Lactiplantibacillus plantarum*) was stored in the laboratory of Northeast Agricultural University (Harbin, Heilongjiang, China). LA (Lactobacillus acidophilus) was stored in the China Microbial Culture Collection Center (Beijing, China). Pepsin (>250 U/mg), trypsin (90 U/mg), and salivary amylase (>5 U/mg) were purchased from Sigma Company (St. Louis, MO, USA). Chromatographic pure methanol, chromatographic pure acetonitrile, chromatographic pure formic acid, chromatographic pure water, and chromatographic pure propanol were purchased from Sigma Company (St. Louis, MO, USA). All kits were purchased from the Nanjing Jiancheng Institute of Bioengineering (Nanjing, Jiangsu, China).

### 2.2. Preparation of Fermented Broad Bean–Mulberry Composite Juice (FBMJ)

Black beans (Manufactured in 2024, Batch No.: HLJ-20240901) were screened to remove defective seeds, with an initial moisture content of (11.8 ± 0.5)% (determined by the GB 5009.3-2016 method) [12]. The dried beans were stored in sealed bags in a cool, dry laboratory area. No mold growth was observed prior to use. This study utilized a total of 2.0 kg of beans from this batch. The mulberry variety (Batch No.: HLJ-20240815) used was black mulberry, with a soluble solids content of (13.2 ± 0.4) °Brix. Samples were stored frozen at −20 °C and thawed at 4 °C for 12 h prior to experimentation, then thoroughly mixed before use. The product was packaged in sterile bags, each containing 1 kg. A total of 3 bags were used in this study. The prepared kidney bean juice and mulberry juice were mixed in a 1:1 (*v*/*v*) ratio to form an unfermented composite raw juice. Analysis showed that this raw juice contained an initial TPC and TFC of (0.91 ± 0.03) mg GAE/mL and (0.52 ± 0.02) mg RE/mL, respectively.

The kidney beans were pulverized in a juicer (XY-8608, Demashi Intelligent Kitchen Equipment Co., Ltd., Guangzhou, Guangdong, China) and mixed at a 1:5 (g/mL) solid-to-liquid ratio. The pH of the bean pulp was adjusted to 6.0. Subsequently, 200 μL of high-temperature α-amylase per 100 mL was added, and the mixture was liquefied for 13 min in a water bath at 95 °C. Afterward, the mixture was cooled to ambient temperature (25 ± 2 °C) and the pH was adjusted to 4.5 (PHST-4F, Ohaus International Trading Co., Ltd., Shanghai, China). A saccharifying enzyme (200 μL/100 mL) was then added, and the mixture was incubated at 70 °C for 25 min. Black bean pulp was obtained by filtering through a mesh screen. Additionally, 2.5% (*w*/*v*) pectinase was added to the mulberry pulp, which was incubated at 45 °C for 30 min. After enzymatic treatment for 30 min, the mixture was filtered through a mesh screen to obtain mulberry juice. The kidney bean turbid juice and mulberry juice were combined in a 1:1 ratio to create a kidney bean–mulberry composite juice, which was supplemented with 6% granulated sugar. Each of the kidney bean juice, mulberry juice, and composite juice samples was sterilized in a pressure cooker (BXM-85BE, Boxun Medical Biological Instrument Co., Ltd., Shanghai, China) at 121 °C for 15 min. The juices were then placed on a clean bench (DL-CJ-2ND, Donglianhal Instrument Manufacturing Co., Ltd., Beijing, China) and fermented in a biochemical incubator (SPX-250Blll, Tester Instruments Co., Ltd., Tianjin, China) for 40 h with 3% mixed lactic acid bacteria (LP:LA = 1:1) to produce a fermented composite juice of kidney beans and mulberries (FBMJ), fermented kidney bean juice (FBJ), and fermented mulberry juice (FMJ). The above findings were obtained through preliminary experiments. For details, please refer to the Supplementary Materials (Figures S1–S3, Tables S1 and S2).

For activation, the lactic acid bacterial strains were inoculated into MRS broth and cultured at 37 °C for 24 h. Cultures were then streaked onto MRS solid plates and incubated for 48 h. Single colonies were selected, inoculated into fresh liquid medium, and incubated for 24 h for further use.

### 2.3. Determination of Bioactive Components During FBMJ Fermentation

#### 2.3.1. Determination of the Total Polyphenol Content (TPC) in FBMJ

According to Zheng et al.’s [13] method, gallic acid was utilized as the standard. A volume of 500 μL of fermentation broth was taken, to which 2.5 mL of Folin–Ciocalteu reagent and 3.75 mL of a 12% sodium carbonate solution were added. The mixture was thoroughly mixed, diluted until the final volume was 10 mL, and allowed to react in the dark for 35 min before measuring the absorbance at 762 nm.

#### 2.3.2. Determination of the Total Flavonoid Content (TFC) in FBMJ

According to Zhang et al.’s [14] method, rutin served as the standard reference substance. After vigorously mixing 500 μL of 7% NaNO_2_ with 1 mL of the FBMJ, the mixture was permitted to stand for 15 min. Subsequently, 500 μL of 15% Al(NO_3_)_3_ was added, and the mixture was shaken vigorously for seven minutes before the addition of 3 mL of 5% NaOH. The final volume was adjusted with 90% ethanol, allowed to stand for 7 min, and the absorbance was then measured at 517 nm.

### 2.4. Analysis of Antioxidant Activity

#### 2.4.1. DPPH Radical Scavenging Ability Assay of FBMJ

The test sample was mixed in a specific ratio with a 0.2 mmol/L DPPH solution (prepared in 95% ethanol), diluted 2-fold with ethanol containing FBMJ, and 1 mL of dilution buffer was added to the system. In the final reaction system, the concentration of FBMJ was approximately 1.67 mg/mL. The solution was reacted in the dark for 45 min, and the absorbance was measured at 510 nm [15]. The calculation formula is as shown in Equation (1):

(1) $$\mathrm{DPPH}(\%)=(1-\frac{A_{1}-A_{2}}{A_{0}})\times 100\%$$

A_0_: absorbance value without sample; A_1_: absorbance value of the sample solution after the color development reaction; and A_2_: absorbance value without the color developer.

#### 2.4.2. Hydroxyl Radical Scavenging Ability Assay of FBMJ

Testing was conducted using the Nanjing Jiancheng Hydroxyl Radical Test Kit, using FBMJ diluted 2-fold with the appropriate assay buffer; then, we added 200 μL of dilution buffer to the system. In the final reaction system, the concentration of FBMJ was approximately 1.67 mg/mL.

#### 2.4.3. ABTS Radical Scavenging Ability Assay of FBMJ

Testing was conducted using the Nanjing Jiancheng ABTS Free Radical Assay Kit, using FBMJ diluted 2-fold with the appropriate assay buffer; then, we added 10 μL of dilution buffer to the system. In the final reaction system, the concentration of FBMJ was approximately 1.25 mg/mL.

### 2.5. In Vitro Gastrointestinal Digestion

#### 2.5.1. Preparation of Simulated Digestive Juices

For the preparation of oral simulated digestive juices, 7500 U of salivary amylase was added to 200 mL of an oral electrolyte solution (composed of 0.75 mmol/L CaCl_2_·2H_2_O, 2 mol/L KCl, and 0.5 mol/L KH_2_PO_4_), stirred for 8 min, and the pH was adjusted to 6.8 using 1 mol/L NaOH.

In the case of gastric simulated digestive fluid, 2 × 10^5^ U of pepsin was added to 200 mL of gastric electrolyte solution (comprising 1 mol/L KCl, 1.5 mol/L NaHCO_3_, 2.5 mol/L NaCl, and 0.3 mol/L CaCl_2_), it was stirred at ambient temperature (25 ± 2 °C) for 8 min, and 1 mol/L HCl was used to get the pH down to 2.0.

Lastly, for intestinal simulated digestive fluid, 5 × 10^4^ U of trypsin was added to 300 mL of intestinal electrolyte solution (which included 20 mmol/L bile salt, 0.25 mol/L KCl, 1.25 mmol/L CaCl_2_·2H_2_O, and 200 U/mL pancreatic juice), mixed at ambient temperature (25 ± 2 °C) for 8 min, and 1 mol/L NaOH was added to bring the pH down to 7.0 [16].

#### 2.5.2. Simulate Oral and Gastric Digestion

According to the method established by Chang et al. [17], the digestion stage in this experiment was divided into three groups: the gastric juice digestion group, the gastric acid control group (which consisted of gastric simulated digestive fluid without the addition of pepsin), and the blank control group (which only included HCl for treatment). To evaluate the impact of digestive enzymes on the rate of active chemical release, the FBMJ was diluted in ratios of 1, 2, and 4. Subsequently, these dilutions were added to the oral digestive fluid in equal proportions. The mixture was oscillated for two minutes at 37 °C and 200 r/min (using the PYPYX-01, PeiYing Experimental Equipment Co., Ltd., Suzhou, China). To complete the oral simulated digestion, 1 mol/L HCl was injected to bring the pH down to 2.0. Following this, 10 mL of gastric juice was added (the fermentation complex juice from the three groups was diluted twice), and the mixture was shaken at 37 °C and 200 r/min. Samples were taken at 0, 30, 60, 90, and 120 min during the gastric digestion period.

#### 2.5.3. Simulated Intestinal Digestion

The intestinal fluid digestion group and the blank control group (intestinal digestion solution without adding trypsin) were added to the gastric digestive fluid. NaOH was injected to adjust the pH level to 6.8, and 10 mL of intestinal fluid (FBMJ of all three groups was diluted twice) was added to the digestion system. After that, the mixture was incubated at 200 rpm at 37 °C. During the stomach digesting phase, samples were collected at 0, 30, 60, 90, and 120 min.

The blank control group and the simulated digestive juices from each stage were immersed in a hot water bath for ten minutes. The resulting products from the simulated gastric digestion (SGKM) group, gastric acid control (SKM) group, simulated intestinal digestion (SIKM) group, and the blank control (BC) group were obtained by centrifuging at 5000 r/min for ten minutes. The total polyphenol content (TPC), total flavonoid content (TFC), and antioxidant capacity were subsequently analyzed.

### 2.6. Non-Targeted Metabolomics Analysis

#### 2.6.1. Sample Preparation

For the analysis, 400 μL of extraction solvent (acetonitrile:methanol, 1:1 *v*/*v*) was added to a 1.5 mL centrifuge tube containing 100 μL of the liquid sample. The sample was then spiked with a mixed internal standard solution (e.g., containing L-2-chlorophenylalanine at 0.02 mg/mL). The mixture was vortexed for 30 s, subjected to ultrasonic extraction at 5 °C and 40 kHz for 30 min, and subsequently kept at −20 °C for 30 min. After centrifugation at 13,000× *g* and 4 °C for 15 min (5430R, Eppendorf AG, Hamburg, Germany), the supernatant was transferred to a new tube. The supernatant was dried under a gentle stream of nitrogen (JXDC-20, Shanghai Jingxin Industrial Development Co., Ltd., Shanghai, China). The residue was reconstituted in 100 µL of a 1:1 (*v*/*v*) acetonitrile/water solution. The reconstituted solution was briefly sonicated at 5 °C and 40 kHz for 5 min (LNG-T88, Taicang Huamei Biochemical Instrument Factory, Taicang, Jiangsu, China) and then centrifuged at 13,000× *g* and 4 °C for 10 min. Finally, the resulting supernatant was carefully transferred to an injection vial equipped with an insert for instrumental analysis.

QC sample processing: To assess the repeatability of the analytical process, quality control (QC) samples were prepared by mixing equal volumes of the metabolite extracts from all experimental samples. In the LC-MS analysis, a total of 21 samples were injected: 18 experimental samples (from six groups) and 3 QC samples.

#### 2.6.2. Instrument and Chromatographic Conditions Setup

The sample extracts were analyzed using Thermo Fisher’s ultra-high-performance liquid chromatography–tandem Fourier transform mass spectrometry UHPLC-Orbitrap Exploris 240 system (Shanghai Meiji Biomedical Technology Co., Ltd., Shanghai, China). Data acquisition was performed in both positive ion (ESI+) and negative ion (ESI−) scanning modes to ensure broad metabolite coverage.

The chromatographic conditions were as follows: After being separated on a column (100 mm × 2.1 mm i.d., 1.8 µm) (HSS T3 Chromatography Column Waters Corporation, Milford, MA, USA), a 3 μL sample was submitted for mass spectrometry. Mobile phase A was a water/acetonitrile (95/5, volume/volume) solution containing 0.1% formic acid, and mobile phase B was an acetonitrile/isopropanol/water (47.5/47.5/5, volume/volume/volume) solution containing 0.1% formic acid. The column temperature was 40 °C, and the flow rate was 0.40 mL/min. Acetonitrile/isopropanol/water was employed as mobile phase B to provide a broader elution range, ensuring effective separation and detection of metabolites across all polarities. All organic solvents used for LC-MS analysis (methanol, acetonitrile, and isopropanol) were chromatographic grade (HPLC grade), formic acid was HPLC grade, and water was LC-MS grade.

The mass spectrometry conditions are as follows: The sample mass spectrometry signal was collected using both positive and negative ion scanning modes with a mass scanning range of m/z: 70–1050 and a resolution of 60,000. The Automatic Gain Control target was set to Standard, and the Maximum Injection Time was 100 ms. Following each full scan, the top 10 most intense ions were selected for tandem mass spectrometry (MS/MS) acquisition. The acquisition mode was DDA. The resolution was set to 15,000, the AGC target was Standard, and Maximum IT was 50 ms. Fragmentation employed high-energy collision dissociation (HCD). The ion beam voltages are as follows: positive ions 3500 V and negative ions −3000 V; the sheath gas was 50 argon; the auxiliary heating gas was 50 argon; the ion source temperature was 350 °C; the normalized collision energy (NCE) was in step mode; and the cycling collision energy was 20–40–60 V.

#### 2.6.3. Data Collection and Metabolite Identification Workflow

Raw mass spectrometry data were processed using Progenesis QI software (v3.0, Waters Corporation). The workflow included peak extraction and alignment, retention time correction, background subtraction, and normalization (based on the total ion current intensity across all samples). Subsequently, extracted characteristic peaks (containing m/z, retention time, and MS/MS spectrum information) were compared against public databases such as the Human Metabolome Database (HMDB, https://hmdb.ca, accessed on 12 July 2025) and METLIN (https://metlin.scripps.edu, accessed on 12 July 2025) for metabolite identification. The identification criteria were set as follows: primary mass spectrometry mass error < 10 ppm, and secondary mass spectrometry fragmentation score > 30. To guarantee data quality, only metabolites with RSD < 30% in positive as well as negative ion mode QC samples were added to later analytical matrices.

This experimental metabolite identification method adhered to the confidence levels defined by the Metabolomics Standards Initiative (MSI). The databases used for metabolite qualitative matching include in-house libraries created from reference standards on the same mass spectrometry platform, as well as mainstream public databases like the HMDB and METLIN. We considered the mass tolerance limits for both parent ions and fragments (error < 10 ppm). Qualitative grade Bi (fragmentation score) was the score obtained by matching against a reference library constructed in-house. Bii (Theoretical Fragmentation Score) was the score obtained by matching against theoretical spectra in public databases such as the HMDB and Metlin. The filtering principle was to retain metabolites with Bi > 35 and Bii > 40. Targeted identifications of compounds were made by comparing them to the running parameters of standards (e.g., retention time, mass number, mass number error, and MS/MS scores) (Table S3).

The qualitative level was determined by Bi (fragmentation score), which was the score obtained from matching against a self-built reference library, and Bii (Theoretical Fragmentation Score), which was the score obtained from matching against theoretical spectra in public databases such as the HMDB and Metlin. Our filtering principle was to retain metabolites with Bi > 35 and Bii > 40. The primary process involved the unambiguous identification of metabolites based on precise mass (error < 10 ppm) and diagnostic MS/MS fragment ion matching, under clearly defined qualitative rating levels. Annotation was inferred through database comparison and classification into presumed metabolite compound categories.

#### 2.6.4. Stability Assessment

This experiment primarily evaluated and ensured system stability through the following aspects. First, the overlap of the total ion chromatograms (TICs) for QC samples was assessed, revealing highly overlapping peak shapes and retention times. This indicates that the chromatographic separation system maintained stability throughout the entire sequence. Subsequently, the stability of the internal standard response was evaluated. Based on the internal standard’s peak intensity over all QC runs, the relative standard deviation (RSD) was computed. The RSD of the internal standard response in both positive and negative ion modes was <15%. Visualization using z-score box plots confirmed the absence of outliers, demonstrating a stable system response. The most critical step involves assessing and correcting the stability of metabolite peak intensities. We calculated the RSD for all metabolite characteristic peaks detected in QC samples. Metabolites with an RSD > 30% in QC samples are typically considered unstable features and were excluded. Finally, within the data analysis software (Progenesis QI), the processing workflow itself incorporated a retention time alignment step to correct for minor retention time drift.

### 2.7. Statistical Analysis

Each experiment was repeated thrice. Intergroup differences were analyzed using one-way ANOVA. If significant overall differences were detected, Tukey’s post hoc test was further applied for multiple comparisons. Plots were generated using Origin 8.0 software. If *p* < 0.05, the results were statistically significant. To validate the preliminary identification of metabolites, an accuracy error of 10 ppm was imposed in both MS and MS/MS data. The gathered data was utilized to determine the chemical structure and name of metabolites using the available online databases, including the HMDB (http://www.hmdb.ca, accessed on 12 July 2025) and Metlin (https://metlin.scripps.edu/, accessed on 12 July 2025). After eliminating peaks with missing values in more than 80% of the data, the remaining matrix was further reduced. The Majorbio Cloud Platform (https://www.majorbio.com, accessed on 12 July 2025), a free online platform, was then utilized to assess the overall difference between groups, utilizing principal component analysis (PCA) and partial least squares discriminant analysis (PLS-DA). The Kyoto Encyclopedia of Genes and Genomes (KEGG) database resource (https://www.kegg.jp, accessed on 5 August 2025) was used to study the metabolic pathway. Venn plot and volcano plot analyses were conducted using the ropls package (Version1.6.2) in the R language. Significant differential metabolites were eliminated using the PLS-DA model’s VIP ≥ 1, *p*-value < 0.05, FC > 2, and FC < 1/2 criterion.

## 3. Results and Discussion

### 3.1. Changes in TPC and TFC During FBMJ Fermentation Process

**Figure 1:**
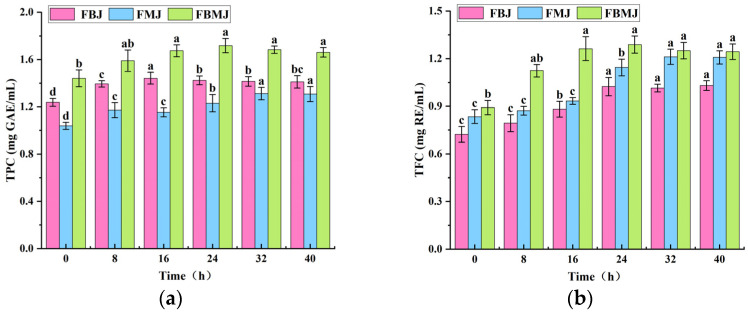

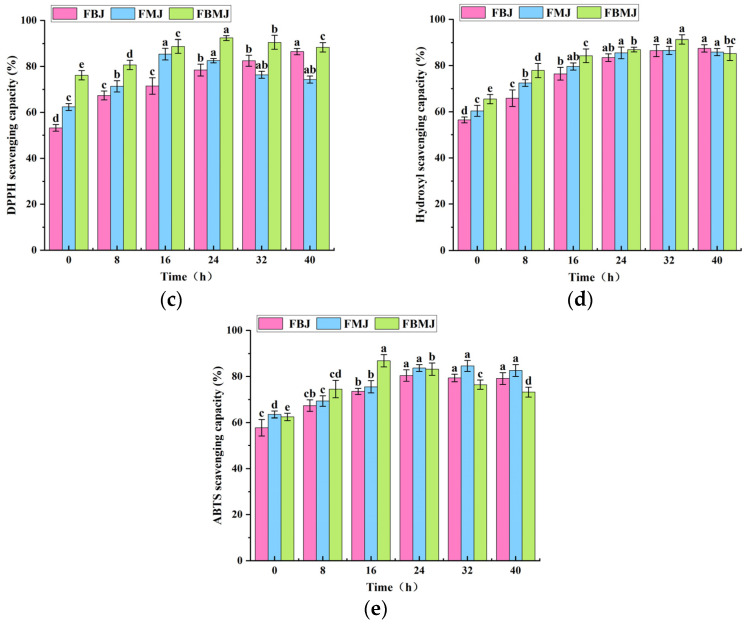
Changes in polyphenol (**a**), flavonoid (**b**), DPPH (**c**), hydroxyl (**d**), and ABTS (**e**) radical scavenging rates content in fermented juice from different raw materials at various fermentation times. FBMJ: kidney bean–mulberry composite fermented juice; FBJ: fermented kidney bean juice; FMJ: fermented mulberry juice. Different letters indicate significant differences (*p* < 0.05) between treatment groups at the same digestion time point.

The findings regarding the TPC and TFC in various fermentation broths are shown in Figure 1a,b. Compared to the FBJ and FMJ groups, the FBMJ group was characterized by higher TPC and TFC, showing that composite fermentation effectively addressed the deficiencies in polyphenol and flavonoid contents. Consequently, the composite fermentation of kidney beans and mulberries selected for this study effectively enhanced the TPC and TFC in the fermentation broth.

TPC and TFC indirectly influence antioxidant activity through structural modifications. As shown in Figure 1a, the TPC concentration in the FBMJ group peaked at 24 h of fermentation, measuring (1.718 ± 0.061) mg GAE/mL, a 1.19-fold increase from the initial level. Figure 1b shows that the TFC concentration in the FBMJ group also peaked at 24 h of fermentation, reaching (1.289 ± 0.053) mg RE/mL, a 1.45-fold increase from the initial level. However, TFC levels began to decline after 24 h of fermentation. These findings are consistent with the research published by Wang et al. [5] and Hu et al. [18]. Kidney beans and mulberries are rich in polyphenols and flavonoids. Lactic acid bacteria partially degrade cell walls through enzymes produced during fermentation, thereby releasing more bound polyphenols and flavonoids. Among these, bound polyphenols and flavonoids undergo structural transformations such as glycoside hydrolysis and methylation, converting from bound to free states, thereby synergistically enhancing antioxidant activity [19,20]. The findings show that structural transformation is the primary factor contributing to the increased TPC and TFC, synergistically enhancing antioxidant activity.

### 3.2. Changes in Antioxidant Activity During FBMJ Fermentation Process

The findings show that the FBMJ group has higher DPPH, ABTS, and hydroxyl radical scavenging rates compared to the FBJ and FMJ groups (Figure 1c,d). Composite fermentation likely stimulates the synthesis of TPC- and TFC-type compounds, which significantly enhances the antioxidant activity of the fermentation broth.

Antioxidant activity is closely associated with TPC and TFC. In the experiment, ascorbic acid served as the positive control. Under the experimental conditions, the scavenging rates of ascorbic acid at a concentration of 0.1 mg/mL were 95.27%, 92.84%, and 95.42% for DPPH, hydroxyl radicals, and ABTS, respectively. Following 32 h of fermentation, the DPPH radical scavenging rate of FBMJ peaked at 94.12% (Figure 1c). The hydroxyl radical scavenging capability exhibited a consistent upward trend, reaching 91.32% (Figure 1d). Figure 1e demonstrates that FBMJ achieved a peak ABTS radical scavenging rate of 83.17% at 16 h of fermentation, reflecting a 0.65-fold increase from the initial fermentation stage. Additionally, these findings were consistent with the research published by Wang et al. [5] and Hu et al. [18]. The two researchers prepared fermented juices using a composite of kidney beans and a composite of mulberries, respectively, both of which resulted in enhanced antioxidant activity. According to previous reports, both kidney beans and mulberries have been extensively demonstrated to possess significant in vitro antioxidant activity. This had been attributed to their rich content of structurally diverse plant secondary metabolites, particularly polyphenolic compounds. Polyphenols, as reducing agents, could directly donate hydrogen atoms or electrons to free radicals, thereby enhancing their scavenging capacity and boosting antioxidant activity. Therefore, the observed increase in DPPH, hydroxyl, and ABTS radical scavenging rates in this study stems from the fermentation process, enhancing both the overall concentration of reductive substances in FBMJ and their reaction efficiency [21]. This enhancement provided greater capacity for hydrogen atom or electron donation, thereby improving the composite’s ability to engage in electron-transfer reactions. The above results showed that FBMJ exhibits enhanced radical scavenging potential after fermentation, providing a chemical basis for subsequent validation of its biological effects in cellular or animal models.

### 3.3. Correlation Analysis

Table 1 shows the correlation study of total phenolic content (TPC), total flavonoid content (TFC), DPPH radical scavenging rate, ABTS radical scavenging rate, and hydroxyl radical scavenging capacity during FKBL fermentation. TPC and TFC in FKBL showed significant positive correlations with antioxidant capacity (*p* < 0.01). This is likely due to the fermentation-induced conversion and release of polyphenols and flavonoids, forming highly active aglycones or phenolic acids, which enhance antioxidant properties. According to the studies, the main antioxidant molecules produced during FKBL fermentation were TPC and TFC chemicals.

### 3.4. Changes in TPC and TFC Release of FBMJ During In Vitro Digestion

**Figure 2:**
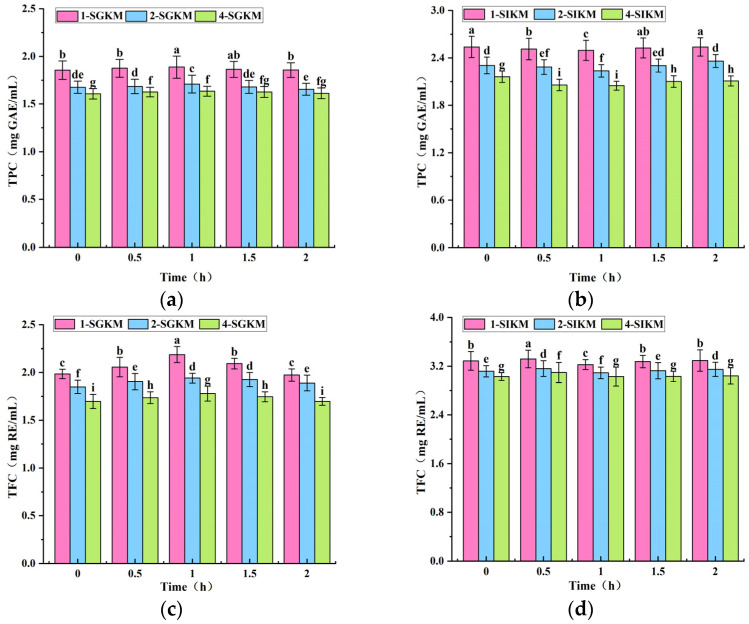

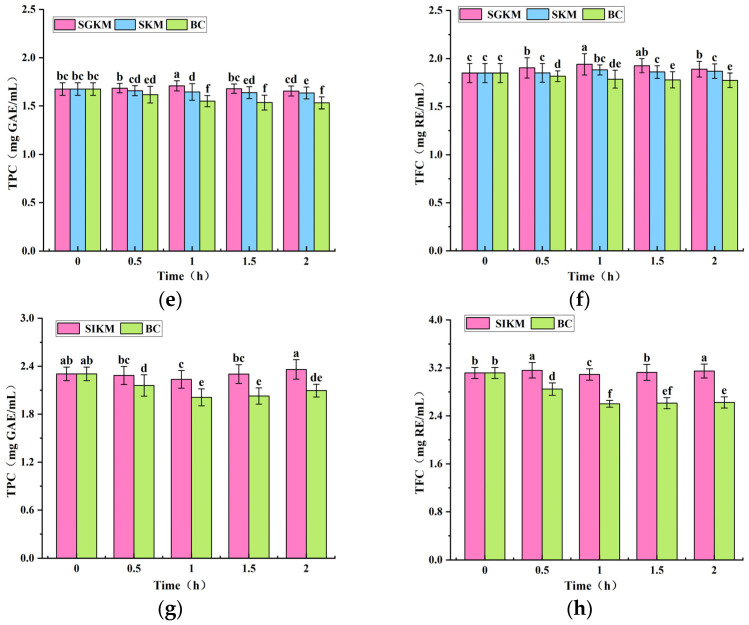
The comparisons of in vitro bioactivities of FBMJ among different groups. (**a**–**d**) The effects of different concentrations of in vitro simulated gastric digestion and intestinal digestion on the release of TPC and TFC in the complex fermentation liquid; (**e**,**f**) the effects of simulated gastric digestion of pepsin on the release of TPC and TFC in complex fermentation broth in vitro; (**g**,**h**) the effects of simulated intestinal digestion of pancreatic enzymes on the release of TPC and TFC in complex fermentation broth in vitro. 1-SGKM: gastric digestion group at 1-fold dilution; 2-SGKM: gastric digestion group at 2-fold dilution; 4-SGKM: gastric digestion group at 4-fold dilution; 1-SIKM: intestinal digestion group at 1-fold dilution; 2-SIKM: intestinal digestion group at 2-fold dilution; 4-SIKM: intestinal digestion group at 4-fold dilution. SGKM: the simulated gastric digestion product; SKM: the gastric acid control group; SIKM: the simulated intestinal digestive product; BC: the blank control group. Different letters indicate significant differences (*p* < 0.05) between treatment groups at the same digestion time point.

The findings show that polyphenols and flavonoids alter the antioxidant activity of FBMJ. To examine changes in its antioxidant activity under in vitro conditions, simulation tests were conducted. By sampling at different time points, temporal variations were monitored to assess the release of polyphenols and flavonoids during digestion. FBMJ was diluted at various concentrations to eliminate concentration interference and the impact of inherent properties. Both the TPC and TFC in FBMJ at various concentrations exhibited an initial increase followed by a decrease during the gastric digestion phase (Figure 2a,b). During the intestinal digestion phase, TPC first reduced then increased, while TFC increased, decreased, and resurged. Significant changes (*p* < 0.05) were observed in TPC and TFC across different dilution levels, showing a continuous delayed release of polyphenols and flavonoids as the digestion time prolonged. Additionally, the TPC and TFC changes in the 2-SGKM and 2-SIKM groups were notably stable, so a 2-fold dilution was selected for subsequent experiments. The concentration trends of polyphenols and flavonoids were relatively comparable during the simulated gastric digestion phase. Previous studies have shown that the release of some phenolic compounds bound to proteins during protein breakdown may be the cause of the rise in TPC [22]. Furthermore, enzymatic digestion and pH changes likely accelerated polyphenols converting from bound to free states. The breakdown of proteins mediated by pepsin results in the liberation of flavonoids from their entrapment in macromolecular protein complexes. The observed substantial increase in TFC is likely explained by this mechanism.

During gastric digestion, the SGKM group showed the highest TPC and TFC, followed by the SKM group, while the BC group was the lowest (Figure 2c,d). Both pepsin and gastric acid stimulated the release of TPC and TFC during digestion, with pepsin having a stronger effect. The SGKM group reached peak TPC and TFC levels at 1 h, with values of (1.710 ± 0.054) mg GAE/mL and (1.941 ± 0.111) mg RE/mL, reflecting increases of 1.17- and 1.38-fold compared to 0 h. In contrast, the SKM group reached peak TPC at (1.646 ± 0.087) mg GAE/mL and TFC at (1.881 ± 0.053) mg RE/mL at 0.5 h, showing increases of 1.03 and 1.04 times, respectively, relative to 0 h. The synergistic action of gastric acid and pepsin likely caused increased TPC. The polyphenols and their complexes are hydrolyzed to release free polyphenols.

The hydrolysis of polyphenols and their complexes by pancreatic enzymes likely caused the release of free phenolic acids (Figure 2e,f). TFC peaked at 0.5 h in both groups, with values of (3.161 ± 0.130) and (2.847 ± 0.103) mg RE/mL, respectively, before declining. This reduction may result from prolonged digestion, possibly hydrolyzing ester molecules or partially degrading flavonoids. Flavonoids initially bound to proteins may have been released through enzymatic digestion, as evidenced by the subsequent increase in flavonoid concentration after one hour [23].

In vitro simulated digestion showed that TPC and TFC increased by 1.42 and 1.66 times, respectively, during intestinal digestion compared to gastric digestion. These results were consistent with the research published by Cele et al. [24], which showed that trypsin can facilitate the conversion of bound polyphenols and flavonoids into free forms. The observed discrepancy in TPC and TFC levels between the end of gastric digestion and the onset of intestinal digestion may result from the partial breakdown of probiotic-rich composite fermentation juice in gastric acid. This breakdown likely decreased initial intestinal digestion concentrations and rapidly adjusted pH to activate pancreatic enzymes and bile salts [25].

### 3.5. Changes in Antioxidant Activity of FBMJ During In Vitro Digestion

**Figure 3:**
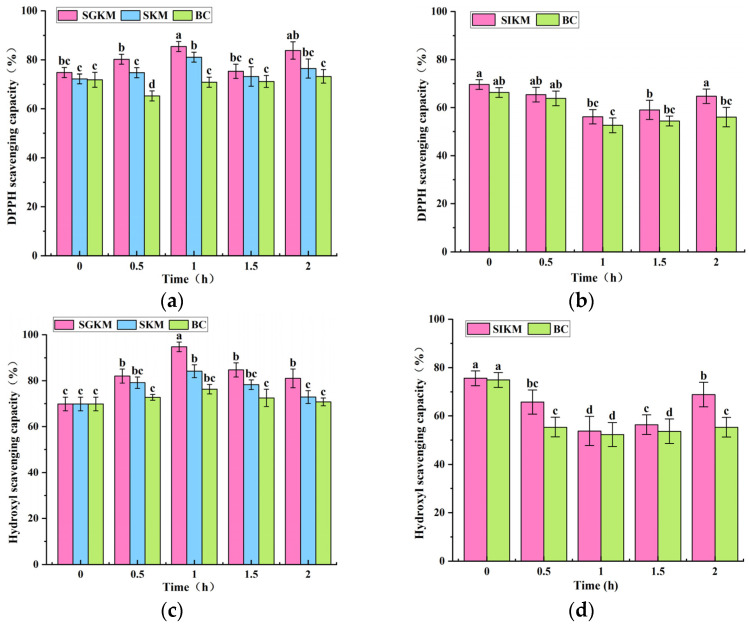

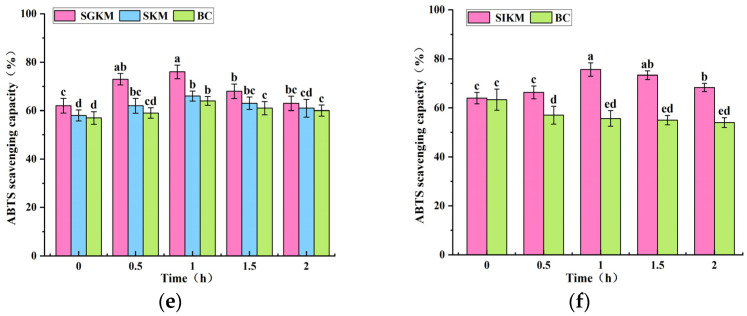
The comparisons of in vitro antioxidant activity of FBMJ among different groups (**a**,**b**) The changes in DPPH radical scavenging rate; (**c**,**d**) the changes in hydroxyl radical scavenging rate; (**e**,**f**) the changes in ABTS radical scavenging rate. Different letters indicate significant differences (*p* < 0.05) between treatment groups at the same digestion time point.

Changes in antioxidant activity in vitro are influenced by the release of polyphenols and flavonoids. The SGKM group exhibited the highest antioxidant activity during the gastric digestion process, followed by the SKM group, and the BC group exhibited the lowest activity (Figure 3). Pepsin and stomach acid in the gastric environment positively enhanced antioxidant capacity. After one hour, the clearance rates of DPPH radicals (Figure 3a,b), hydroxyl radicals (Figure 3c,d), and ABTS radicals (Figure 3e,f) in the SGKM group increased by 1.22, 1.36, and 1.14 times, respectively. In the SKM group, these rates also peaked at one hour, increasing by 1.12, 1.14, and 1.22 times, respectively. During the intestinal digestion stage, the scavenging rates of DPPH free radicals and hydroxyl radicals initially decreased before increasing. However, no significant difference was observed in the scavenging rate of DPPH free radicals, which reached minimal values of 56.19% and 52.62% at one hour, respectively. One hour post-digestion, the scavenging rate of ABTS free radicals began to decline after achieving its peak at one hour, increasing by 1.18 times. Additionally, compared to the BC group, the SIKM group demonstrated enhanced scavenging abilities for DPPH radicals, ABTS radicals, and hydroxyl radicals. Research has shown that pancreatic enzymes enhance the scavenging ability for ABTS radicals and hydroxyl radicals, but do not significantly affect the scavenging ability for DPPH radicals.

These findings were in agreement with the research undertaken by Nignpense et al. [26]. We confirmed that phenolic components play a significant role in the radical scavenging process of FBMJ. It is noteworthy that antioxidant activity during the intestinal digestion phase was lower than that observed during the gastric digestion phase. This discrepancy may be attributed to the influence of the chemical conditions in the intestinal environment on phenolic compounds. In this environment, it can impede the passive diffusion and absorption of phenolic compounds through epithelial cells. By failing to effectively contact and react with free radicals, it limits their radical scavenging capacity, thereby reducing antioxidant activity during the intestinal phase. According to previous research reports, the acid-base environment and enzyme activity during digestion influenced the efficiency with which phenolic compounds donated hydrogen atoms, thereby regulating their radical scavenging capacity [27]. This shows that the pH and enzymatic activity environment at each stage of digestion influence the antioxidant properties of phenolic compounds. To gain a more systematic understanding of how the digestive process affects antioxidant activity, future research should focus on analyzing the structural transformation patterns of key phenolic compounds under simulated gastrointestinal conditions. This should be combined with evaluations of their absorption and antioxidant effects using intestinal epithelial cell models.

However, it should be noted that the antioxidant activity generated by this system may result from multiple combined factors. As a rich source of protein, kidney beans inevitably release peptides under the action of pepsin and trypsin. Current research shows that kidney bean protein hydrolysates and their isolated peptides exhibit significant in vitro antioxidant activity. The antioxidant mechanisms of these peptides may exhibit complementary or synergistic effects with the detected phenolic compounds. Future research will explore the effects of peptide substances on antioxidant activity, systematically analyzing their relative contributions and potential synergistic effects post-digestion.

### 3.6. Non-Targeted Metabolomics Analysis During Fermentation and In Vitro Digestion Stages

**Figure 4:**
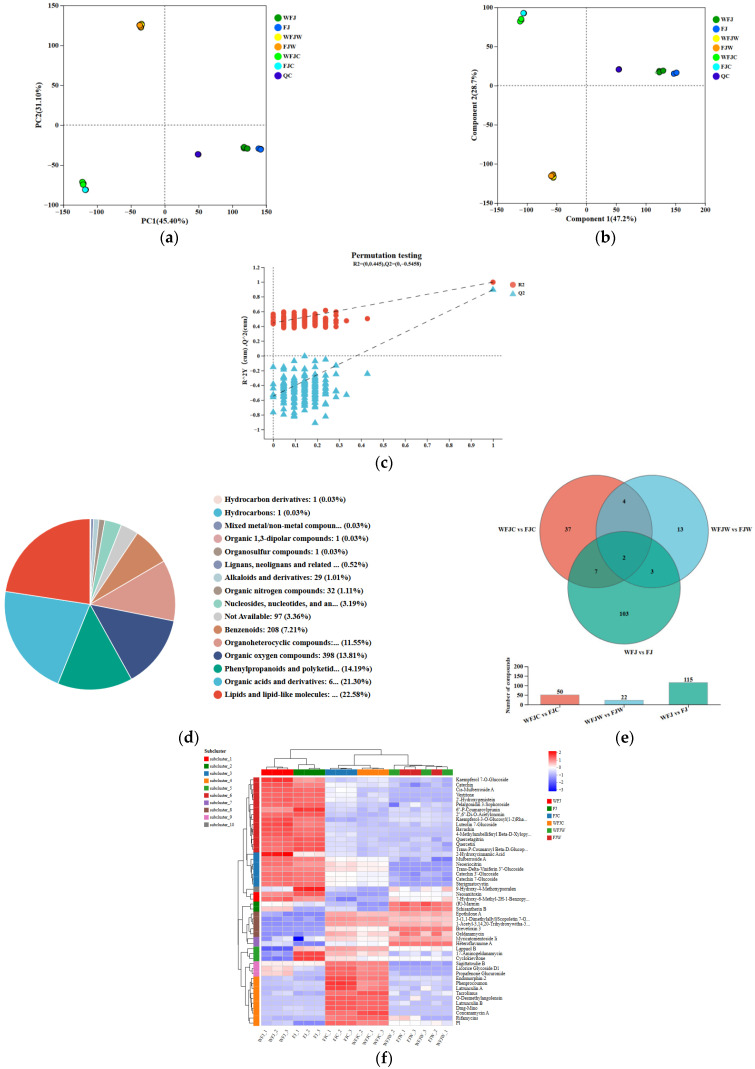
The multivariate and cluster analysis of PPE at different stage. PCA score plot (**a**–**c**); HMDB heatmap (**d**); Venn heatmap (**e**); HCA heatmap (**f**). The colors of the heatmap represent the relative amount of each metabolite, with green to red showing low to high levels. WFJ: unfermented group; FJ: Fermentation Completion Group; WFJW: pre-gastric digestion group; WFJ: post-gastric digestion group; WFJC: pre-digestive intestinal group; FJC: post-intestinal digestion group.

The PCA score plot shows the variance among samples within groups and highlights the overall metabolic differences between groups. According to the PCA results, PC1 accounted for 45.40% of the variance, while PC2 contributed 31.10%, showing significant variations in FBMJ across different stages (Figure 4a). Sample similarity and intergroup differences were visually represented using partial least squares discriminant analysis (PLS-DA) (Figure 4b). A PLS-DA replacement test was conducted to assess the model’s validity and reliability. The samples demonstrated good grouping with minimal intra-group heterogeneity (Figure 4c). The decreasing R2 and Q2 values showed an intercept of Q^2^ < 0.5 and *p* < 0.05, demonstrating that the developed model successfully avoided overfitting. This underscores the stability and dependability of the PLS-DA model. Although PLS-DA showed distinct separation in metabolite distribution across different groups, these findings must be validated with univariate statistical tests (such as t-tests and fold-change values) to confirm differential metabolites, thus avoiding false positive conclusions resulting from overfitting.

The Human Metabolome Database (HMDB, https://hmdb.ca, accessed on 12 July 2025) is recognized as the most comprehensive database of human-specific metabolites and is often referred to as the “gold standard” in the field of metabolomics (Figure 4d). This database encompasses over 200,000 metabolites and provides a variety of analytical methods like metabolic pathway analysis and metabolite enrichment analysis. By aligning the metabolite classification information obtained from the HMDB database, we identified a total of 2883 metabolites across 16 categories. The predominant groups included lipids and lipoid molecules (22.58%), organic acids and their derivatives (21.30%), phenylpropanoids and polyketides (14.19%), organic oxides (13.81%), and organic heterocyclic compounds (11.55%).

Furthermore, our study has established optimal fermentation conditions for producing a fermented composite juice that enriches polyphenolic components. Given that the biological activity of FBMJ may be influenced by the types and quantities of polyphenolic compounds, we further investigated the profiles of polyphenolic components throughout the fermentation and digestion stages of FBMJ.

The Venn diagram showed that the total numbers of metabolites in the WFJ vs. FJ, WFJW vs. FJW, and WFJC vs. FJC groups were 115, 22, and 50, respectively. This reflected significant metabolic variations in the polyphenolic components of FBMJ before and after fermentation, with changes occurring as a result of gastrointestinal digestion (Figure 4e). A total of 115 polyphenolic compounds were identified during the fermentation phase, 22 during gastric digestion, and 50 during intestinal digestion. Abundance variations in secondary metabolites across groups were clearly depicted in the cluster heatmap (Figure 4f). Notably, 22 groups showed significantly higher abundance from fermentation to digestion, while 27 groups showed significantly lower abundance (*p* < 0.05).

### 3.7. Differential Analysis of FBMJ Phenolic Metabolites at Different Stages During Fermentation and In Vitro Digestion Stages

**Figure 5:**
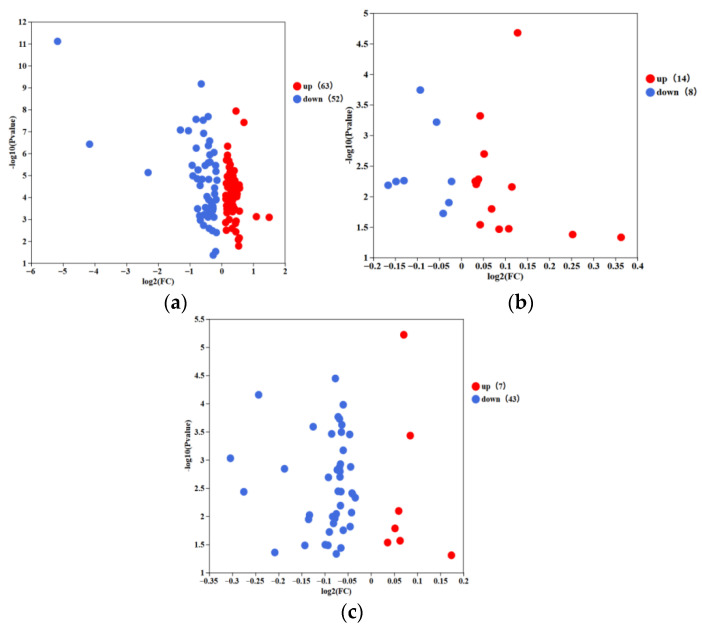
Volcano plot analysis of differential phenolic metabolites in FBMJ at different stages. (**a**) The volcano plot analyzes metabolites before and after fermentation; (**b**) the volcano plot analyzes metabolites before and after gastric digestion; (**c**) the volcano plot analyzes metabolites before and after intestinal digestion.

Significantly different metabolites were screened based on the PLS-DA model’s criteria: *p*-value < 0.05, VIP ≥ 1, and fold-change FC > 2 or FC < 1/2 [10]. Volcano plots showed variable metabolite expression levels across distinct FBMJ groups (Figure 5a–c). The results showed that during the fermentation stage, 63 polyphenolic metabolites were elevated while 52 were downregulated. In the stomach digestion stage, 14 metabolites were elevated and 8 were downregulated. Throughout the intestinal digestion stage, 7 metabolites were elevated and 43 were downregulated. Studies have shown that from fermentation to simulated gastric and intestinal digestion, the number of differentially upregulated metabolites has steadily decreased, whereas significantly downregulated metabolites have progressively increased.

**Figure 6:**
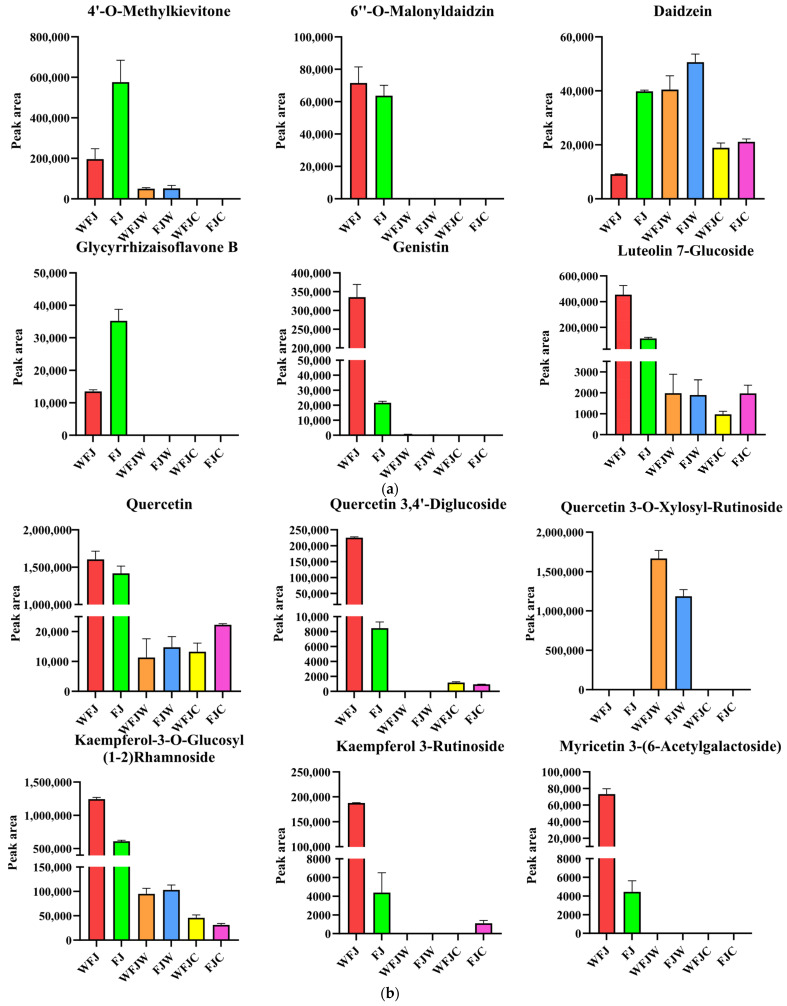

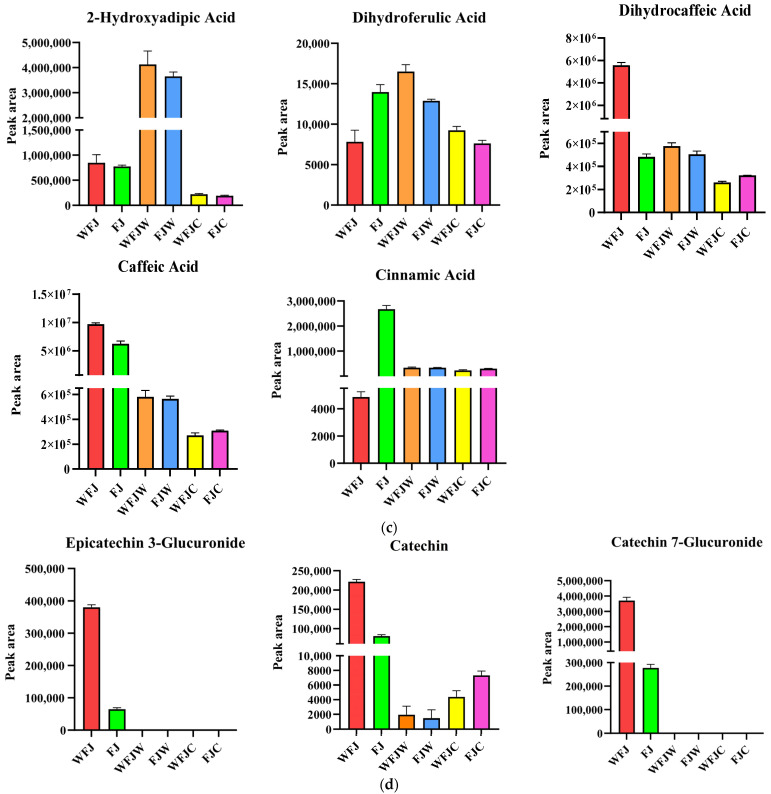
Changes in the abundance of FBMJ phenolic compounds at different stages. (**a**) Isoflavones; (**b**) flavonols and their glycosides; (**c**) phenolic acids; (**d**) flavan-3-ols.

During the fermentation to digestion stage of FBMJ, based on mass spectrometry profiling, we detected a total of 20 polyphenolic metabolites showing significant changes in abundance (Table 2). The first group includes isoflavonoids (Figure 6a). During fermentation, 6″-O-malonylgenistein, genistin, 4′-O-methylkykonin, daidzein aglycone, and glycyrrhizin B’s relative abundance showed increasing trends. In the digestion phase, 4′-O-methylkykonin and daidzein aglycone’s relative abundance showed declining trends. Based on the previous literature reports, both genistein and daidzein in this study were substances with well-established antioxidant activity. The conversion of low-activity conjugated isoflavone precursors into highly active aglycones (such as genistein and daidzein) and specifically modified derivatives during fermentation might be associated with the enhanced antioxidant activity in the system [28,29]. Additionally, the partially methylated derivatives remain relatively stable in the environment during the digestion phase and may exert a persistent antioxidant effect in the body through absorption [30]. The findings showed that genistein and daidzein are associated with variations in antioxidant activity and may be key compounds responsible for differences in antioxidant potency.

The second group includes flavanols and their glycosides (Figure 6b). During fermentation, quercetin-3,4′-diglucoside, quercetin-3-O-xylosyl-rutinoside, kaempferol-3-O-glucosyl-(1-2)-rhamnoside, kaempferol-3-rutinoside, and myricetin-3-(6-acetylgalactoside)’s relative abundance showed a declining trend, whereas quercetin’s relative abundance demonstrated an increasing trend. In the digestion phase, quercetin-3-O-xylosyl-rutinoside and kaempferol-3-O-glucosyl-(1-2)-rhamnoside’s relative abundance displayed a decreasing trend, while quercetin-3,4′-diglucoside and kaempferol-3-rutinoside’s relative abundance showed an upward trend. Previous studies have confirmed that quercetin possesses potent antioxidant activity. The antioxidant mechanism of quercetin encompasses not only direct radical scavenging but also, more significantly, its iron ion chelation capability and the Nrf2 signaling pathway’s activation [31]. Their increased relative abundance correlates with enhanced antioxidant capacity. These changes may collectively regulate the antioxidant properties of the final product both inside and outside the body.

The third group includes phenolic acids (Figure 6c). During fermentation, dihydroferulic acid and cinnamic acid’s relative abundance increased, while 2-hydroxycinnamic acid, dihydroxycinnamic acid, and caffeic acid’s relative abundance showed a declining trend. Previous studies have shown that the conversion products of phenolic acids exhibit significant radical scavenging capacity [32]. However, the overall decrease in abundance during the digestion phase shows that these substances may undergo significant transformation or degradation within the gastrointestinal environment. Previous studies have reported that cinnamic acid and its derivatives, such as ferulic acid, are released after oral digestion and degraded during gastrointestinal digestion [33]. Therefore, cinnamic acid was not the main reason for the antioxidant activity.

The fourth group includes flavan-3-ols (Figure 6d). Throughout fermentation, epicatechin-3-glucuronide, catechin, and catechin-7-glucuronide’s relative abundance showed a declining trend; however, catechin’s relative abundance showed an increasing trend during digestion. Previous studies have confirmed that catechins are widely recognized as effective antioxidants. Furthermore, the findings of this study align with previous literature reports, indicating that fermentation and digestion enhance bioavailability by converting bound forms into free catechins [34,35]. The findings show the key mechanism by which catechins enhance antioxidant activity.

The results further confirm that phenolic compounds are associated with changes in antioxidant activity. These results are consistent with the research published by Jiang et al. [36]. The research findings show that during fermentation and digestion, daidzein, genistein, quercetin, and catechin are associated with changes in antioxidant activity, and may be the potential compounds contributing to the enhanced antioxidant activity of FBMJ. These findings provide theoretical mechanisms and clues for subsequent research on compound-specific targeting technologies.

### 3.8. Analysis of Key Bioactive Compound Levels and Metabolic Pathways

**Figure 7:**
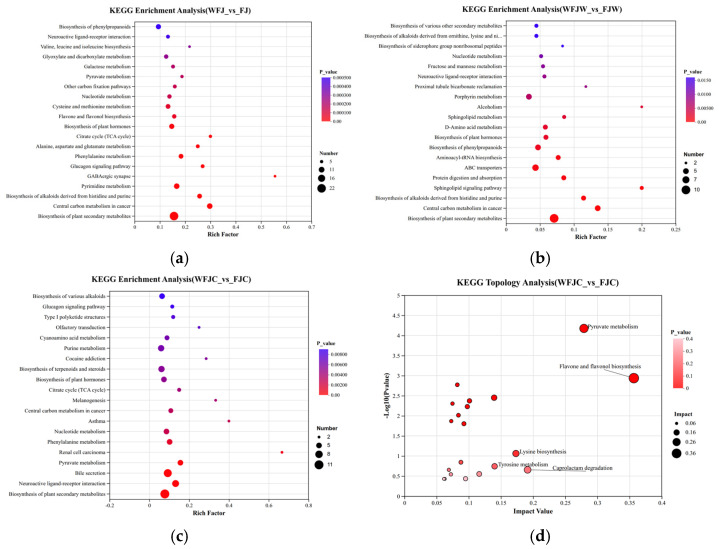
KEGG pathway enrichment analysis of differentially metabolized polyphenols across comparison groups. (**a**–**c**) Correspond to the top four most significantly enriched pathways in WFJ vs. FJ (pre- vs. post-fermentation), WFJW vs. FJW (pre- vs. post-gastric digestion), and WFJC vs. FJC (pre- vs. post-intestinal digestion), respectively. (**d**) KEGG topology analysis diagram. Bubble size indicates the proportion of enrichment (larger indicates greater enrichment); bubble color indicates the significance of enrichment (closer to red indicates more significant).

To systematically elucidate the potential associations among group-specific differential metabolites, we performed KEGG pathway enrichment analysis (Figure 7a–d and Table 3). The results showed that a total of 51 differential metabolites were significantly enriched in the synthetic pathway categories of the synthesis of plant secondary metabolites, biosynthesis of phenylpropanoid compounds, biosynthesis of flavonoids and flavanols, and phenylalanine metabolism. It classified most key differential metabolites at the compound level as phenolic compounds, particularly flavonoids and their precursors. Among these, quercetin, genistein, and their respective precursors (quercetin 3-O-sophoroside, 6″-O-malonylgenistein) and their upstream amino acid precursors (L-tyrosine, L-phenylalanine) were significantly enriched in the flavonoid and flavanol biosynthesis pathways categories. These findings show that the significant enrichment of phenolic metabolites in the flavonoid and flavanol biosynthetic pathways may be associated with alterations in antioxidant activity. The analysis results strongly support the aforementioned conclusions of this study. The enhanced antioxidant activity of FBMJ is primarily attributed to the efficient conversion of the polyphenolic compounds from bound to free states and from glycoside to aglycone forms. KEGG analysis not only validates the taxonomic classification of core metabolites at the chemical taxonomy level but also provides a theoretical basis for explaining their synergistic transformation from fermentation to digestion.

## 4. Conclusions

This study investigated the use of a composite lactic acid bacteria strain to ferment a juice blend consisting of kidney beans and mulberries. This study investigated the antioxidant activity mechanism from fermentation to digestion through in vitro simulated digestion and non-targeted metabolomics analysis, examining changes in TPC, TFC, and free radical scavenging capacity. The results show that the co-fermentation of kidney beans and mulberries with composite strains enhanced their polyphenol and flavonoid content and enhanced free radical scavenging capacity, which contributed to increased antioxidant activity. The potential contribution of protein-derived bioactive peptides, though not characterized in this study, warrants further investigation. The antioxidant activity of FBMJ is primarily influenced by polyphenolic compounds. In vitro digestion facilitated the release of TPC and TFC, resulting in increased antioxidant activity. Non-targeted metabolomics studies show that during fermentation and digestion, daidzein, genistein, quercetin, and catechin may be the potential compounds contributing to the enhanced antioxidant activity of FBMJ. According to the KEGG metabolic pathway analysis, phenolic metabolites are significantly enriched in the biosynthetic pathways of flavonoids and flavanols. This study investigates the patterns of antioxidant changes and provides a fundamental mechanism analysis. It lays the groundwork for further research into the potential roles of these antioxidant components within complex biological systems, thereby advancing related applied studies. Future research will build upon the findings of this study in the following ways: Validating the antioxidant activity of the identified key compounds individually and in combination using cellular oxidative stress models. Conducting animal studies to assess the bioavailability and in vivo efficacy of these phenolic compounds from FBMJ. Identifying and characterizing the peptide fraction generated during digestion to evaluate its potential synergistic or additive effects with phenolic compounds.

## Figures and Tables

**Table 1:** Correlation between TPC, TFC and antioxidant indexes of KBMJ.

	TPC	TFC	DPPH Radical	ABTS Radical	Hydroxyl Radical
TPC	1	0.980 **	0.948 **	0.966 **	0.849 *
TFC	/	1	0.876 **	0.933 **	0.857 *
DPPH radical	/	/	1	0.916 *	0.798
ABTS radical	/	/	/	1	0.713
Hydroxyl radical	/	/	/	/	1

** *p* < 0.01 (highly significant difference); * *p* < 0.05 (significant difference).

**Table 2:** Relative abundance of polyphenolic compounds in FBMJ samples across groups.

Category	Compound Name	Peak Area					
		WFJ	FJ	WFJW	FJW	WFJC	FJC
Isoflavones	4′-O-Methylkievitone	196,296	576,560	50,541.1	51,906.2	ND	ND
	6″-O-Malonyldaidzin	71,562.3	63,702.8	ND	ND	ND	ND
	Daidzein	9140.58	39,849	40,488.8	50,634.1	18,938.4	21,130
	Glycyrrhizaisoflavone B	13,517.6	35,246.8	ND	ND	ND	ND
	Genistin	335,410	21,690.2	423.9	212.1	ND	ND
	Catechin 7-Glucuronide	3,699,861	277,704	600.1	ND	ND	ND
Flavonols and their glycosides	Quercetin	1,605,236	1,416,980	11,321.5	14,736.1	13,283.9	22,279.1
	Quercetin 3,4′-Diglucoside	225,121	8455.5	ND	ND	1178.9	950.19
	Quercetin 3-O-Xylosyl-Rutinoside	ND	ND	1,665,126	1,185,245	ND	ND
	Kaempferol-3-O-Glucosyl(1-2)Rhamnoside	1,242,876	609,979	94,920.5	103,114	45,927.3	31,209.3
	Kaempferol 3-Rutinoside	187,668	4389	ND	ND	ND	1101.62
	Myricetin 3-(6-Acetylgalactoside)	73,199	4453.3	ND	ND	ND	ND
Phenolic acids	2-Hydroxyadipic Acid	846,117	773,055	4,124,921	3,652,320	219,539	191,635
	Dihydroferulic Acid	7832.4	13,972.4	16,503.8	12,883.7	9236.9	7629.7
	Dihydrocaffeic Acid	5,576,307	481,852	575,895	505,112	260,045	321,914
	Caffeic Acid	ND	ND	580,710	564,862	270,279	308,102
	Cinnamic Acid	4859.5	2,675,515	343,680	344,440	234,658	307,348
Flavan-3-ols	Epicatechin 3-Glucuronide	379,792	64,214.3	ND	ND	ND	ND
	Catechin	221,410	80,347.4	1963.6	1493.6	4390.5	7319.7
	Catechin 7-Glucuronide	3,699,858	277,704	600.1	ND	ND	ND

**Table 3:** Metabolic pathways of polyphenolic compounds in FBMJ.

	Pathway	Match Status	Enrich Factor		
			WFJ vs. FJ	WFJW vs. FJW	WFJC vs. FJC
a	Biosynthesis of plant secondary metabolites	22/175	0.1560	0.0709	0.0780
b	Biosynthesis of phenylpropanoids	10/175	0.0943	0.0472	ND
c	Flavone and flavonol biosynthesis	8/175	0.1569	ND	ND
d	Phenylalanine metabolism	9/175	0.1837	0.0408	0.1020

## Data Availability

The original contributions presented in the study are included in the article/Supplementary Materials, further inquiries can be directed to the corresponding author.

## Supplementary Materials

The following supporting information can be downloaded at: https://www.mdpi.com/article/10.3390/foods15060991/s1, Figure S1: Effects on live bacteria count and SOD activity in FKML, Figure S2: Effects of interaction of various factors on viable count and SOD activity, Figure S3. Changes in reducing sugar and soluble solid content during fermentation process; Table S1: Results of regression analysis of viable count activity model and regression coefficient, Table S2: Results of regression analysis of SOD activity model and regression coefficient, Table S3: Identification Table for Phenolic Metabolites with Significant Abundance.

## Abbreviations

The following abbreviations are used in this manuscript:

FBMJ	Kidney bean–mulberry composite fermented juice
FBJ	Fermented kidney bean juice
FMJ	Fermented mulberry juice
SGKM	Simulated gastric digestion product
SKM	Gastric acid control
SIKM	Simulated intestinal digestive product
BC	Blank control
TPC	Total polyphenol content
TFC	Total flavonoid content
PCA	Principal component analysis
PLS-DA	Partial least squares discriminant analysis
KEGG	Kyoto Encyclopedia of Genes and Genomes
HMDB	Human Metabolome Database
