# Supplementary material for: Study on the Antioxidant Activity of Fermented Broad Bean–Mulberry Composite Juice Based on In Vitro Digestion and Non-Targeted Metabolomic Analysis
Source: Foods. 2026 Mar 11;15(6):991. doi: 10.3390/foods15060991 (PMC13024891; doi:10.3390/foods15060991)
Supplement: Supplementary file 1 [file foods-15-00991-s001.zip › foods-4092734-supplementary.pdf]

3. Results and discussion

3.1 Single-factor and Response Surface Optimization of Process Conditions for FKML

3.1.1 Single-Factor Experimental Analysis

The SOD activity and viable cell count showed an upward trend at pH 4.0-5.0. The SOD activity and viable bacterial count peaked at pH 5.0, reaching 288.9 U/mL and 28.467 × 10^8 CFU/mL, respectively. Beyond pH 5.0, both SOD activity and viable bacterial count began to decline (Figure S1a).

The SOD activity and viable bacterial count reached their maximum values at a raw material mixing ratio of 1:1, at 318.8 U/mL and 29.933 × 10^8 CFU/mL, respectively. An appropriate raw material ratio is more conducive to bacterial growth. A 1:1 mixture of kidney beans and mulberries is more favorable for lactic acid bacteria proliferation, thereby promoting the production of various secondary metabolites by these bacteria (Figure S1b).

At an inoculum level of 3%, SOD activity and viable cell count reached their maximum values of 338.8 U/mL and 25.167 × 10^8 CFU/mL, respectively (Figure S1c).

At a fermentation temperature of 37 °C, SOD activity and viable cell count reached their maximum values of 243.3 U/mL and 24.633×10^8 CFU/mL, respectively, before beginning to decline (Figure S1d).

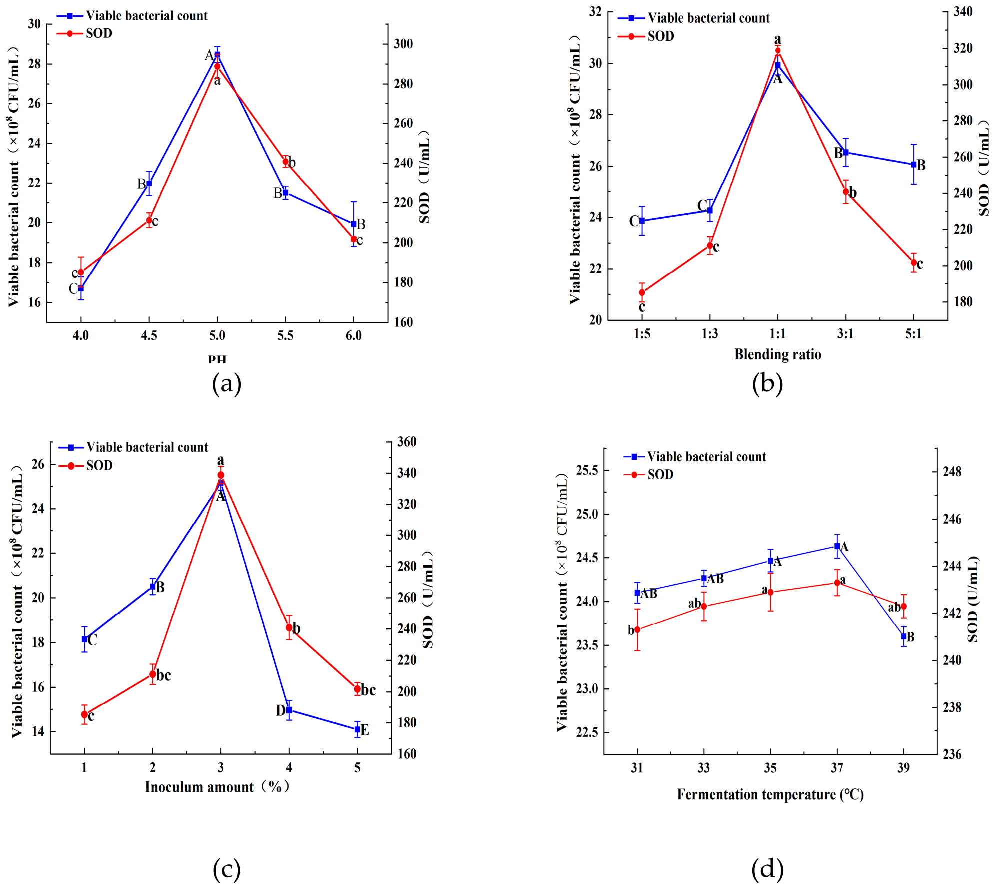

Figure S1. Effects on live bacteria count and SOD activity in FKML. (a): pH; (b): Blending rain; (c): inoculum amount; (d): Fermentation temperature.

3.1.2 Response Surface Experiment Results and Analysis

Based on the results of single-factor experiments, a response surface experiment was designed. With pH (A), blending ratio (B), inoculum amount (C), and fermentation temperature (D) as independent variables, and viable cell count and SOD activity as response values, a four-factor, three-level experimental model was established using the Box-Behnken experimental design method in Design-Expert 13 software.

Regression analysis of the experimental data yielded the quadratic multiple regression equations for viable cell count and SOD activity: $Y_{\text{viable bacterial count}}=28.08+1.30A+1.03B+1.63C+0.70D+0.78AB+0.10AC+2.28AD+0.90BC+1.83BD+0.20CD-4.08A^2-3.03B^2-3.80C^2-2.18D^2$

$Y_{SOD}=363.40-8.03A+6.74B+8.70C+5.27D-5.40AB-13.40AC+15.75AD+0.73BC+11.10BD-1.83CD-25.47A^2-19.11B^2-18.33C^2-7.59D^2$

Table S1. Results of regression analysis of viable count activity model and regression coefficient

Source	Sum of Squared Deviations	Degree of freedom	Mean Square	F-value	P-value	Significance
Model	305.48	14	21.82	8.23	0.0002	**
A-pH	20.28	1	20.28	7.65	0.0152	*
B-blending ratio	12.81	1	12.81	4.83	0.0452	*
C-inoculum amount	32.01	1	32.01	12.08	0.0037	**
D-fermentation temperature	5.88	1	5.88	2.22	0.1585	
AB	2.40	1	2.40	0.9064	0.3572	
AC	0.0400	1	0.0400	0.0151	0.9040	
AD	20.70	1	20.70	7.81	0.0143	*
BC	3.24	1	3.24	1.22	0.2875	
BD	13.32	1	13.32	5.03	0.0417	*
CD	0.1600	1	0.1600	0.0604	0.8095	
A^2	107.84	1	107.84	40.69	<0.0001	**
B^2	59.45	1	59.45	22.43	0.0003	**
C^2	93.79	1	93.79	35.39	<0.0001	**
D^2	30.76	1	30.76	11.60	0.0043	**
residual	37.11	14	2.65			
fictitious term	33.12	10	3.31	3.32	0.1293	ns
pure error	3.99	4	0.9970			
total	342.59	28				
		$R^2=0.8917$	$RAdj^2=0.7834$			

(** P < 0.01 as highly significant, * P < 0.05 as significant, and ns denotes, P > 0.05 as not significant.)

This model exhibits extremely significant differences ($P < 0.0001$); the lack of fit term is insignificant ($P > 0.05$), indicating minimal model error (Table S1). $R^2=0.8917$ demonstrates that the model clearly reflects the fitted experimental results; $RAdj^2=0.7834$ indicates that this model can be used to predict and analyze viable cell counts. Analysis of variance data revealed extremely significant differences in inoculum amount and quadratic terms A^2, B^2, C^2, D^2 ($P < 0.01$), while initial pH, compounding ratio, BD, and AD showed significant differences ($P < 0.05$). The factors influencing viable cell count, in descending order, were C > A > B > D, inoculum amount > pH > compounding ratio > fermentation temperature.

Table S2. Results of regression analysis of SOD activity model and regression coefficient

Source	Sum of Squared Deviations	Degree of freedom	Mean Square	F-value	P-value	Significance
Model	11359.65	14	811.40	6.94	0.0004	**
A-pH	774.41	1	774.41	6.62	0.0221	*
B-blending ratio	545.40	1	545.40	4.66	0.0487	*
C-inoculum amount	908.28	1	908.28	7.76	0.0146	*
D-fermentation temperature	333.91	1	333.91	2.82	0.1133	
AB	116.64	1	116.64	0.9970	0.3350	
AC	718.24	1	718.24	6.14	0.0266	*
AD	992.25	1	992.25	8.48	0.0114	*
BC	2.10	1	2.10	0.0180	0.8953	
BD	492.84	1	492.84	4.21	0.0593	
CD	13.32	1	13.32	0.1139	0.7408	
A^2	4209.57	1	4209.57	35.98	<0.0001	**
B^2	2369.43	1	2369.43	20.25	0.0005	**
C^2	2178.20	1	2178.20	18.62	0.0007	**
D^2	373.43	1	373.43	3.19	0.0957	*
residual	1637.93	14	117.00			
fictitious term	741.37	10	74.14	0.3308	0.9290	ns
pure error	896.56	4	224.14			
total	12997.58	28				
		R^2=0.8740	$RAdj^2$=0.7480			

(** $P < 0.01$ as highly significant, * $P < 0.05$ as significant, and ns denotes, $P > 0.05$ as not significant.)

The model exhibits highly significant differences ($P < 0.0001$), while the non-significant terms ($P > 0.05$) indicate minimal model error (Table S2). With R^2=0.8740, the model clearly reflects the fitted experimental results; $RAdj^2$=0.7480 confirms its suitability for predicting and analyzing SOD. Analysis of variance data revealed that quadratic terms A^2 and B^2 significantly influenced the results ($P < 0.01$), while initial pH, compound ratio, inoculum amount, AC, and AD showed significant effects ($P < 0.05$). The influence of factors on viable cell count ranked as C > A > B > D, inoculum amount > pH > compound ratio > fermentation temperature.

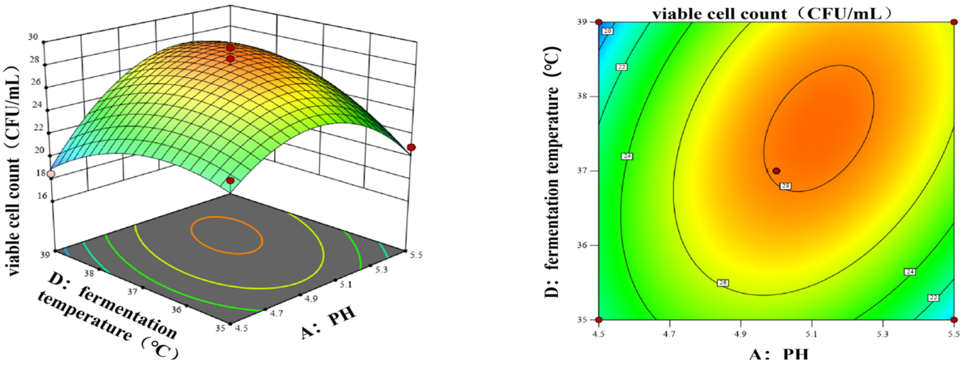

(a)

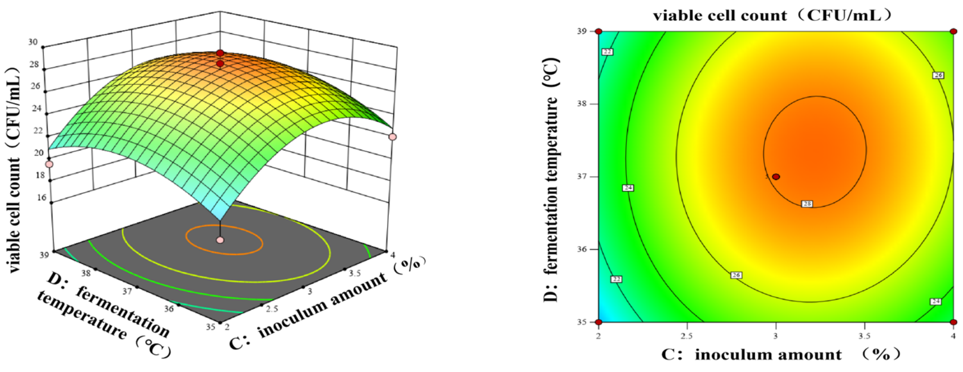

(b)

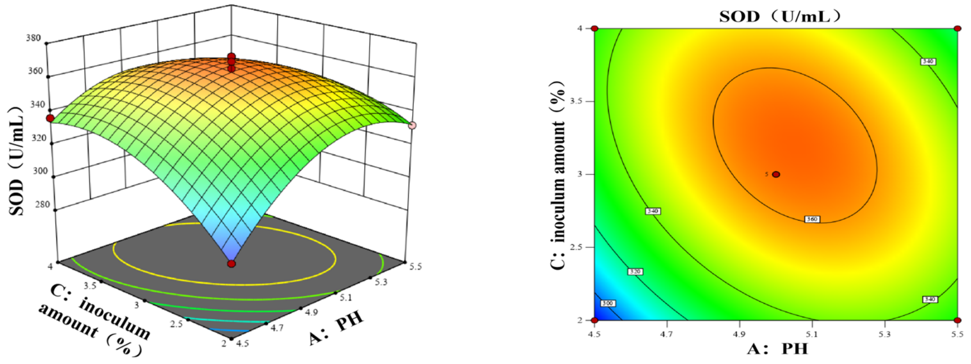

(c)

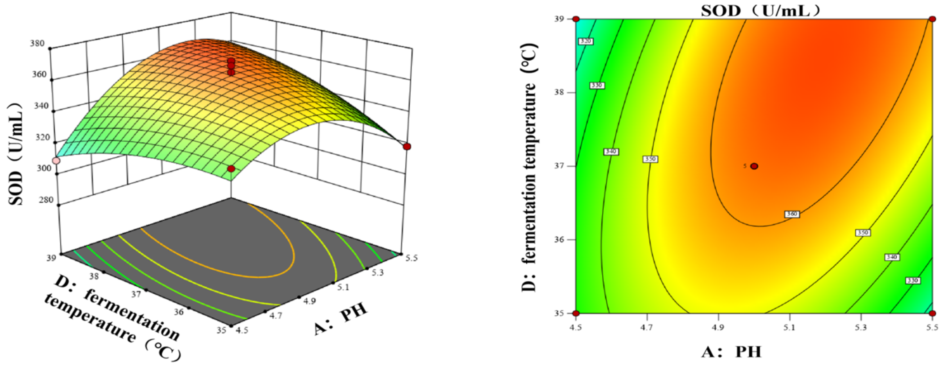

(d)

Figure S2. Effects of interaction of various factors on viable count and SOD activity. (a): viable cell count-AD; (b) viable cell count-cD; (c): SOD-AC; (d): SOD-AD.

3.1.3 Optimal process and validation test results

Optimizing for maximum viable cell count and SOD activity, Design Expert software determined the optimal conditions as follows: pH 5.02, blending ratio 1:1, inoculum level 2.85%, fermentation temperature 36.64 °C. Predicted values are 29.5 × 10^8 CFU/mL for viable cell count and 372.5 U/mL for SOD activity. The optimal fermentation parameters were determined as: initial pH 5.0, compound ratio 1:1, inoculum volume 3%, fermentation temperature 37 °C. Under these conditions, validation measurements yielded a live bacterial count of (26.98 ± 0.63) × 10^8 CFU/mL and SOD activity of (357.49 ± 4.00) U/mL. The actual values fell within a 5% deviation range of the model predictions, indicating high reliability of the process parameters optimized by this model.

3.2 Changes in pH and total acid content during FKML fermentation

Fluctuations in pH are a key indicator of fermentation progress, while changes in total acidity serve as a crucial reference for assessing fermentation broth quality. As fermentation time increased, pH exhibited a downward trend, dropping to 3.66 by 40 hours. Total acidity, however, showed an upward trend, rising from 0.21 mg/mL at 0 hours to 0.78 mg/mL. This indicates that the acidic characteristics of the fermentation broth gradually intensified as fermentation progressed (Figure S3a).

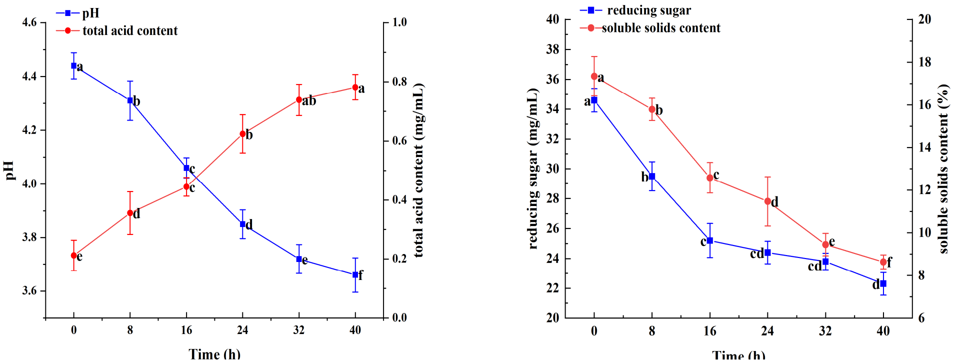

(a) (b)

Figure S3. Changes in reducing sugar and soluble solid content during fermentation process. (a): reducing sugar; (b): soluble solid content. Different letters indicate significant differences (P < 0.05) between treatment groups at the same digestion time point.

3.3 Changes in reducing sugar and soluble solids content during FKML fermentation

Sugar, as a key carbon source in microbial fermentation, reflects microbial activity in the fermentation broth through changes in its concentration. Soluble solids content is a critical factor determining the taste, flavor, and overall quality of the fermentation broth. As fermentation time extended, reducing sugar content gradually decreased, reaching (22.32 ± 0.76) mg/mL at 40 hours of fermentation. Soluble solids content dropped to (8.64 ± 0.33)% after 40 hours of fermentation (Figure S3b).

3.4 Metabolite identification table based on high-resolution mass spectrometry technology

The primary databases were the Human Metabolome Database (HMDB, https://hmdb.ca), METLIN (https://metlin.scripps.edu), and our in-house database.

Table S3. Identification Table for Phenolic Metabolites with Significant Abundance

ID	Metabolite	Metab ID	Library ID	level	KEGG Compound ID	M/Z	Retention time	Mode	Adducts	Formula	Fragmentation Score	Theoretical Fragmentation Score	Mass Error	CAS ID	RSD
pos_18335	4'-O-Methylkievitone	meta b_16686	HMDB0038114	B(ii)	-	335.1277	2.7802	pos	M+H-2H2O	C21H22O6	0	75.7	-0.228538051	_;	0.01235562708659 3
neg_10264	6''-O-Malonyldaidzin	meta b_28591	HMDB0041263	B(ii)	C16191	501.1035	5.2311	neg	M-H	C24H22O12	0	54.3	-0.633987326	124590-31-4	0.02619759700409 2

neg_9816	Daidzein	metab_28176	HMDB0003312;LMPK12050038	B(i)	C10208	253.0502	5.5349	neg	M-H	C15H10O4	41.8	0	-1.818242502	486-66-8;	0.00937094028239996
neg_1272	Glycyrrhizaisoflavone B	metab_20077	HMDB0036556	B(ii)	-	387.0833	1.8863	neg	M+Na-2H	C21H18O6	0	49.8	-4.762498645	197304-07-7	0.039704742599691
pos_4452	Genistin	metab_4017	-;HMDB0033988	B(i)	C09126;_	433.113	3.5261	pos	M+H	C21H20O10	95.8	0	0.190255657243014	529-59-9	0.024572859667282
neg_3574	Catechin 7-Glucuronide	metab_22255	HMDB0127727	B(ii)	-	447.0929	3.7059	neg	M-H2O-H	C21H22O12	0	64.3	-0.746714242	1146696-34-5	0.00999616677005585
pos_6674	Quercetin	metab_6057	HMDB0005794;LMPK12110004	B(i)	C00389	303.0495	4.5175	pos	M+H	C15H10O7	98.9	0	-1.440129917	117-39-5;	0.0168736850292778
neg_3119	Quercetin 3,4'-Diglucoside	metab_21835	HMDB0037363;LMPK12112104	B(i)	-	625.1404	3.4285	neg	M-H	C27H30O17	69.6	0	-1.045070345	29125-80-2;	0.0158749560669777
pos_20767	Quercetin 3-O-Xylosyl-Rutinoside	metab_18906	HMDB0301695	B(ii)	-	765.1781	0.4677	pos	M+Na, M+K	C32H38O20	0	62.5	-9.116285232	129235-39-8	0.2304163089477 8
neg_3144	Kaempferol-3-O-Glucosyl(1-2)Rhamnoside	metab_21859	HMDB0040475	B(i)	-	593.1505	3.4444	neg	M-H	C27H30O15	77.4	0	-1.119279985	142451-65-8	0.00219752657412 98
neg_12454	Kaempferol 3-Rutinoside 4-Glucoside	metab_30671	HMDB0303609	B(i)	-	755.2032	3.7113	neg	M-H	C33H40O20	91.8	0	-1.08861895	-	0.0233362230045 18
neg_5124	Myricetin 3-(6-Acetylgalactoside)	metab_23702	HMDB0035462	B(ii)	-	503.0826	4.6925	neg	M-H2O-H	C23H22O14	0	62	-0.891327977	-	0.0510918253494 37
pos_967	2-Hydroxyadipic Acid	metab_849	HMDB0000321;LMFA01170049	B(i)	C02360	217.0681	0.6535	pos	M+CH3OH+Na	C6H10O5	45.9	0	-0.87288902	-;18294-85-4;	0.0120546514074 14
neg_4712	Dihydroferulic Acid	metab_23321	HMDB0062121	B(i)	-	195.066	4.399	neg	M-H	C10H12O4	43.6	0	-1.237605094	1135-23-5	0.0324818738183 59

pos_1316	Dihydrocaffeic Acid	metab_1121	HMDB0000423	B(i)	C10447	165.0544	1.1059	pos	M+H-H2O	C9H10O4	86	0	-1.44157031	1078-61-1	0.0035006125475209
neg_3806	Caffeic Acid	metab_22474	-;HMDB0001964;HMDB0003501;HMDB0001964	B(i)	C01197;C01481	179.0347	3.8337	neg	M-H	C9H8O4	98.9	0	-1.524628848	501-16-6;331-39-5	0.0068950287084464
neg_11083	Cinnamic Acid	metab_29370	HMDB0000567;HMDB0000930;HMDB0000567;HMDB0000930	B(i)	C10438	147.0449	4.5803	neg	M-H	C9H8O2	56.7	0	-1.508080576	621-82-9;102-94-3;140-10-3	0.0057806534876449
neg_12387	Epicatechin 3-Glucuronide	metab_30606	HMDB0240435	B(ii)	-	465.1034	3.7539	neg	M-H	C21H22O12	0	40	-0.98669741	1146961-52-5	0.009378118513206
neg_12403	Catechin	metab_30621	-;HMDB0002780	B(i)	C06562;C17590	289.0714	3.7486	neg	M-H	C15H14O6	95.7	0	-1.091940514	-;154-23-4	0.0036894999247156
neg_3574	Catechin 7-Glucuronide	metab_22255	HMDB0127727	B(ii)	-	447.0929	3.7059	neg	M-H2O-H	C21H22O12	0	64.3	-0.746714242	1146696-34-5	0.0099961667700585
